# Investigation of a bimetallic terbium(III)/copper(II) chemosensor for the detection of aqueous hydrogen sulfide

**DOI:** 10.3762/bjoc.20.237

**Published:** 2024-11-05

**Authors:** Parvathy Mini, Michael R Grace, Genevieve H Dennison, Kellie L Tuck

**Affiliations:** 1 School of Chemistry, Monash University, Clayton, Victoria 3800, Australia; 2 CBRN Defence, Sensors and Effectors Division, Defence Science and Technology Group, Edinburgh, SA, 5111, and Fishermans Bend, VIC, 3207, Australia

**Keywords:** chemosensor, hydrogen sulfide, lanthanide, luminescence, terbium

## Abstract

The chemosensor properties of a bimetallic terbium(III)/copper(II) complex functionalized with a 4-(2-pyridyl)-1,2,3-triazole ligand for the detection of Cu^2+^ ions and, aqueous and gaseous hydrogen sulfide was investigated. The 4-(2-pyridyl)-1,2,3-triazole ligand functions both as an antenna chromophore and a receptor for Cu^2+^ ions; the Cu^2+^ complex was shown to be a chemosensor for the detection of aqueous hydrogen sulfide. The chemosensor exhibited significant reversibility over multiple cycles, observed with the sequential addition of Na_2_S followed by Cu^2+^ ions. The limit of detection for aqueous hydrogen sulfide was 0.63 μM (20 ppb). No luminescent changes of the bimetallic terbium(III)/copper(II) complex were observed in the presence of gaseous hydrogen sulfide, and thus this sensor can only be used for the detection of aqueous hydrogen sulfide.

## Introduction

The field of luminescent lanthanide chemosensors is rapidly evolving, driven by the need for more efficient, sensitive, and versatile detection methods for environmentally and biologically relevant analytes. While significant advances have been made, there remain critical challenges and unmet needs that call for innovative approaches. One of the key motivations for this exploration is the increasing complexity and diversity of analytes that require detection in real-world scenarios. Traditional methods, while effective, often fall short in environments where multiple, overlapping signals or low concentrations are present. The emerging strategies discussed in this article aim to overcome these limitations by leveraging novel materials, advanced synthesis techniques, and cutting-edge detection mechanisms for the detection of hydrogen sulfide. Hydrogen sulfide (H_2_S) is now recognised as a significant gaseous signaling molecule, alongside nitric oxide and carbon monoxide; it belongs to the biologically active group known as "gaseous mediators" or "gasotransmitters" [1]. In mammalian systems, H_2_S is predominantly biosynthesized at low concentrations through enzymatic conversions of sulfur-containing substrates, and it exerts diverse biological roles across nearly all organ systems. Within the central nervous system, H_2_S functions as a neuromodulator, influencing pain perception and neuronal potentiation [2]. H_2_S is implicated in various pathological conditions such as Parkinson's disease, Alzheimer's disease, Down's syndrome, and diabetes [3–5].

H_2_S naturally occurs in groundwater, originating from the breakdown of organic matter and as a by-product of numerous industrial processes. H_2_S predominantly exists as HS^−^ in the aqueous state at a pH of 7.4 due to its weak acidic nature and high solubility in water (80 mM at 37 °C) [6]. Elevated levels of H_2_S in groundwater pose high risks to both human health and aquatic ecosystems [7], compelling rigorous monitoring of water sources. Even though sensors for detecting aqueous H_2_S are in development [8–14], lack of sensitivity, selectivity, and cost effectiveness remain major challenges.

For a number of years we have explored the properties of lanthanide-based chemosensors (Ln = Eu^3+^ or Tb^3+^) due to their significant advantages over fluorescent-based sensors; notable features include large Stokes shifts, extended luminescent lifetimes, and precisely defined emission bands [15]. Typically lasting in the order of milliseconds, their extended luminescent lifetimes enable the implementation of time-gated detection methods, effectively eliminating short-lived background fluorescence. This unique capability enhances the sensitivity and reliability of trivalent lanthanide-based chemosensors, making them invaluable tools for our application in the detection of hydrogen sulfide.

**Figure 1:**
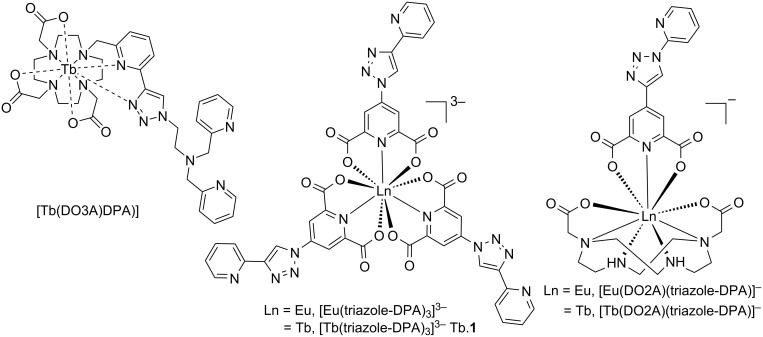
Previously reported lanthanide complexes [12,16–17].

Our previously reported trivalent lanthanide-based chemosensors for the detection of both gaseous and/or aqueous H_2_S are shown in Figure 1 [12,16–17]. These sensors all function via the copper sequestration mechanism, where upon addition of hydrogen sulfide to the quenched bimetallic species, luminescence modulation occurs. In our quest for highly selective, highly sensitive chemosensors via a facile synthetic route/method, we have explored three chelates for lanthanide ions (DO3A, 2,6-pyridinedicarboxylic acid and DO2A), resulting in complexes with different overall charges. Additionally we have explored two copper(II) binding groups (di(2-picolyl)amine and 4-(2-pyridyl)-1,2,3-triazole). A europium(III)/copper(II) complex [Eu(triazole-DPA)_3_·3Cu]^3+^(Figure 1), functionalized with 4-(2-pyridyl)-1,2,3-triazole serving as both an antenna chromophore and a receptor for Cu^2+^ ions, previously demonstrated theoretical limits of detection (LoD) of 1.1 μM for aqueous hydrogen sulfide and 100 ppb for gaseous hydrogen sulfide [16]. However, due to the limited aqueous solubility and ligand dissociation of this chemosensor, and to the weakly luminescent bis species at usable concentrations, we extended this work to the lanthanide–macrocycle binary complexes [Ln(DO2A)(triazole-DPA)·Cu]^+^ (Ln = Eu and Tb, Figure 1). We found that both sensors gave good sensitivity for detection of aqueous H_2_S, however, only the europium variant, [Eu(DO2A)(triazole-DPA)·Cu]^+^_,_ gave a luminescent increase in the presence of gaseous H_2_S. Exposure of [Tb(DO2A)(triazole-DPA)·Cu]^+^ to H_2_S gas resulted in no modulation of luminescent intensity. With this in mind, it was therefore of interest to investigate the luminescent properties of [Tb(triazole-DPA)_3_·3Cu]^3+^, Tb.**1**·Cu, in the presence of aqueous and gaseous hydrogen sulfide.

## Results and Discussion

**Scheme 1:**
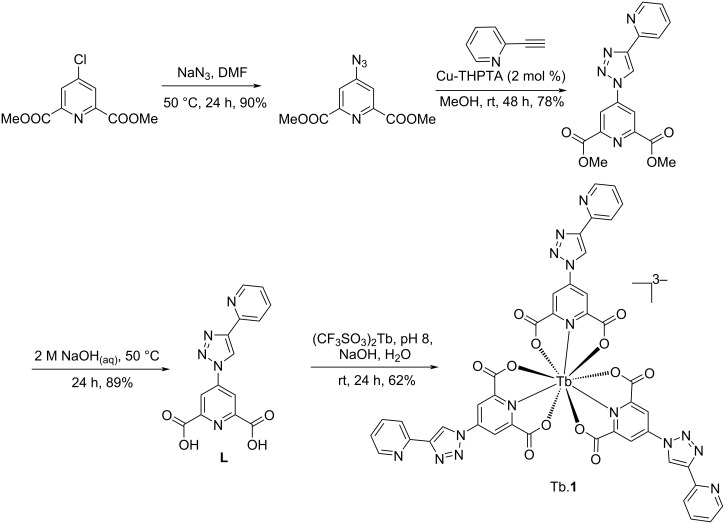
Synthesis of Tb.**1**.

The Tb.**1** complex was synthesized in an analogous fashion to the Eu.**1** complex, via a three-step synthesis as reported previously [16] and depicted in Scheme 1. The corresponding terbium(III) species was synthesized by the combination of three equivalents of the ligand **L** with terbium(III) trifluoromethanesulfonate under basic conditions. High-resolution mass spectrometry (HRMS) analysis and the ^1^H NMR spectrum were consistent with formation of the Tb.**1** complex (Figures S1 and S2 in Supporting Information File 1).

### Luminescence characterization of Tb.**1**

**Figure 2:**
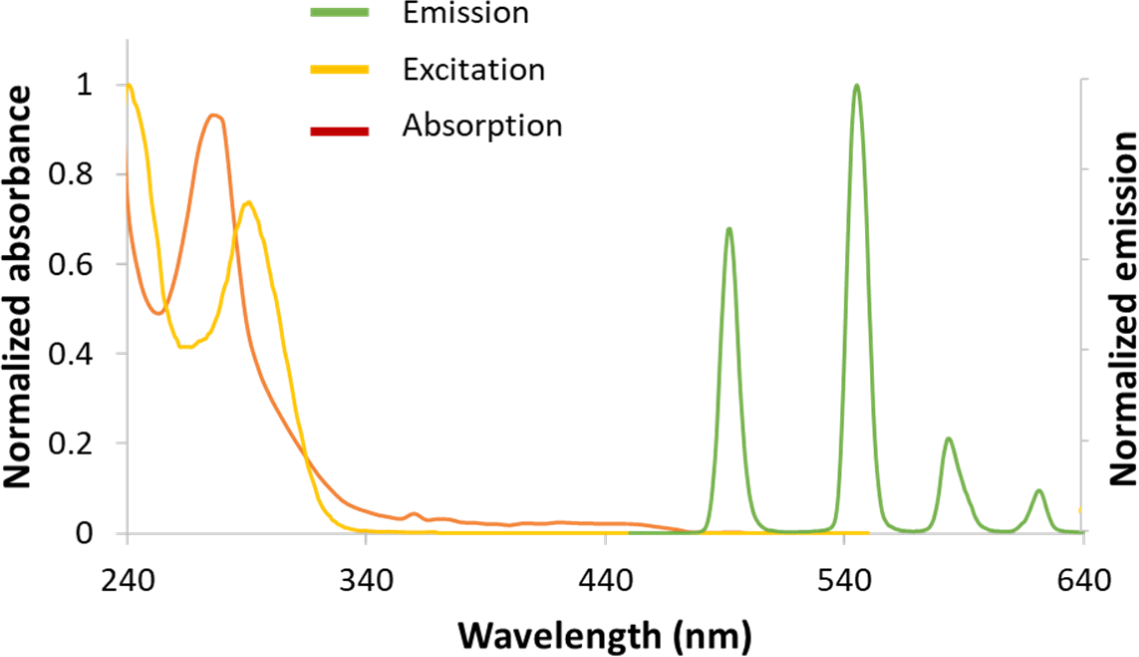
Absorption (red line), excitation (yellow line, λ_em_ = 615 nm), and emission (green line, λ_ex_ = 250 nm) spectra of 5 µM Tb.**1** in 10 mM Tris-HCl buffer, pH 7.4, containing 5% DMSO.

As anticipated, based on the previously reported europium complex ([Eu(triazole-DPA)_3_]^3−^, Tb.**1** exhibited limited solubility in water, becoming insoluble at concentrations exceeding 100 μM. Therefore, a 1 mM stock solution of Tb.**1** was prepared in DMSO, with the final concentration of DMSO in analytical solutions kept at ≤5%. Initial luminescence analysis of a 5 μM solution of Tb.**1** exhibited high luminescence (with a quantum yield of 68%) and displayed the characteristic trivalent terbium emission bands with emission peaks at 491 nm, 545 nm, 583 nm, and 621 nm, corresponding to transitions from the ^5^D_4_ excited state to the ^7^F_6_, ^7^F_5_, ^7^F_4_, and ^7^F_3_ ground states, respectively (Figure 2).

### Response of Tb.**1** with Cu^2+^ ions

The addition of excess Cu^2+^ ions to Tb.**1** was expected to result in complete saturation of emission signals. However, upon the titration of Cu^2+^ ions to 5 μM Tb.**1** (10 mM Tris-HCl buffer containing 5% DMSO, pH 7.4), emission signal saturation was not observed until 5 equivalents (25 μM) were added (Figure S3 in Supporting Information File 1). The addition of 5 equivalents of Cu^2+^ ions led to 70% reduction in the luminescence signal, though complete quenching of luminescence could not be achieved, even with additional Cu^2+^ ions. However, as greater than 50% of emission quenching had occurred when three equivalents of Cu^2+^ ions were added, a [Tb(triazole-DPA)_3_·3Cu]^3+^ [Tb.**1**·3Cu]^3+^ complex was used for the subsequent HS^−^ sensing experiments. We did extend our study to investigate the luminescent quenching of 5 μM Tb.**1** upon the addition of Cu^2+^ ions when 10 mM HEPES buffer containing 5% DMSO, pH 7.4 was used (Figure S4 in Supporting Information File 1), with a similar degree of quenching to that observed in 10 mM Tris-HCl buffer. The incomplete quenching in luminescence was previously also observed for the Eu(III)/Cu(II) complex [16].

Supramolecular.org [18], an Open Access program, was used to determine the binding constant of Cu^2+^ ions to Tb.**1** in both Tris-HCl buffer and HEPES buffer. The host–guest binding modes (1:1, 1:2 or 2:1) were evaluated using the luminescent data (λ_ex_ = 250 nm, λ_em_ = 450–650 nm) from the respective titration experiments. In both cases the 1:1 host–guest binding model gave an acceptable fit with low (co)variance of the fit (Table 1).

**Table 1:** Binding constant determination for the 1:1 host–guest interaction (Tb.**1** + Cu^2+^ ions), determined using supramolecular.org [18].

buffer	binding model (host–guest)	*K (*M^−1^)

10 mM Tris-HCl	1:1	7.4 × 10^4^ M^−1^ ± 0.2%
10 mM HEPES	1:1	9.7 × 10^4^ M^−1^ ± 0.1%

### Aqueous buffer HS^−^ studies of [Tb.**1**·3Cu]^3+^

**Figure 3:**
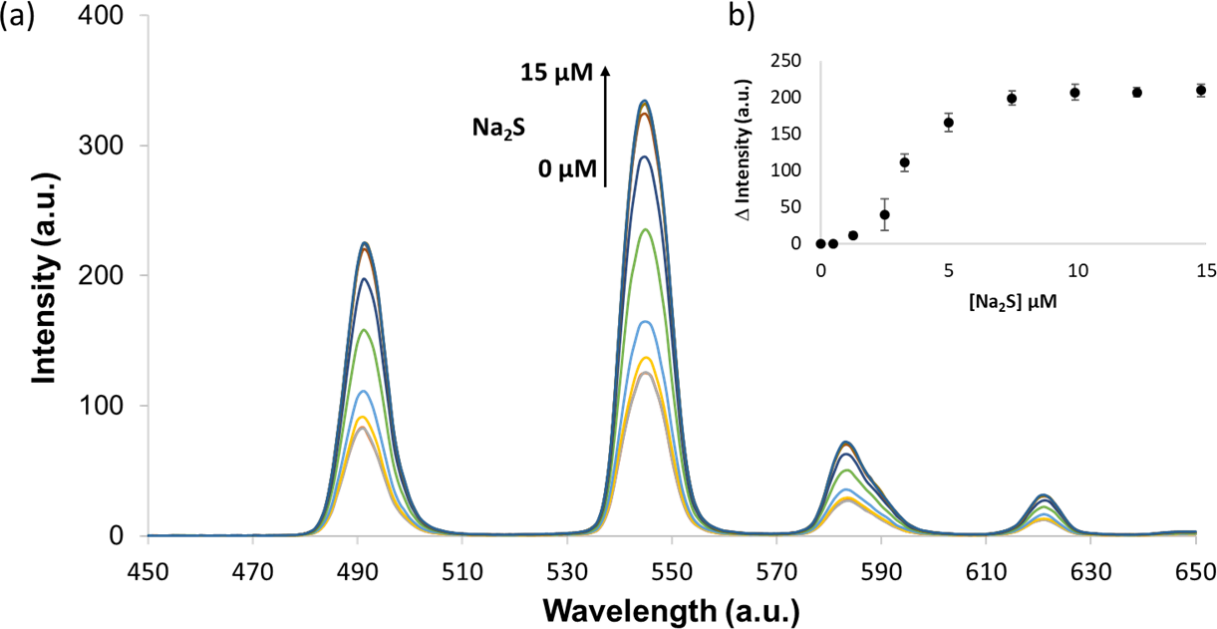
(a) Changes in the luminescence emission spectrum of 5 µM [Tb.**1**·3Cu]^3+^ upon the addition of Na_2_S (0–15 µM); spectra measured in 10 mM HEPES buffer (pH 7.4) with λ_ex_ = 250 nm. (b) Change in luminescence intensity detected at 545 nm upon the addition of Na_2_S (*n* = 3).

The response of [Tb.**1**·3Cu]^3+^ to Na_2_S (HS^−^ in solution at pH 7.4) in both 10 mM HEPES and 10 mM Tris-HCl was investigated. The addition of HS^−^_(aq)_ ions to a solution of [Tb.**1**·3Cu]^3+^, 10 mM HEPES buffer (pH 7.4) resulted in a sigmoidal growth curve, with a “lag phase” (Figure 3). This was an unexpected result as a linear increase in luminescence regain was anticipated on addition of HS^−^_(aq)_ ions to the solution. We interpret this “lag phase” as a consequence of Cu^2+^ ions present in solution, whereby the added HS^−^_(aq)_ ions are mostly consumed by the unbound Cu^2+^ ions. This leads to a gradual luminescence increase rather than the anticipated linear response.

**Figure 4:**
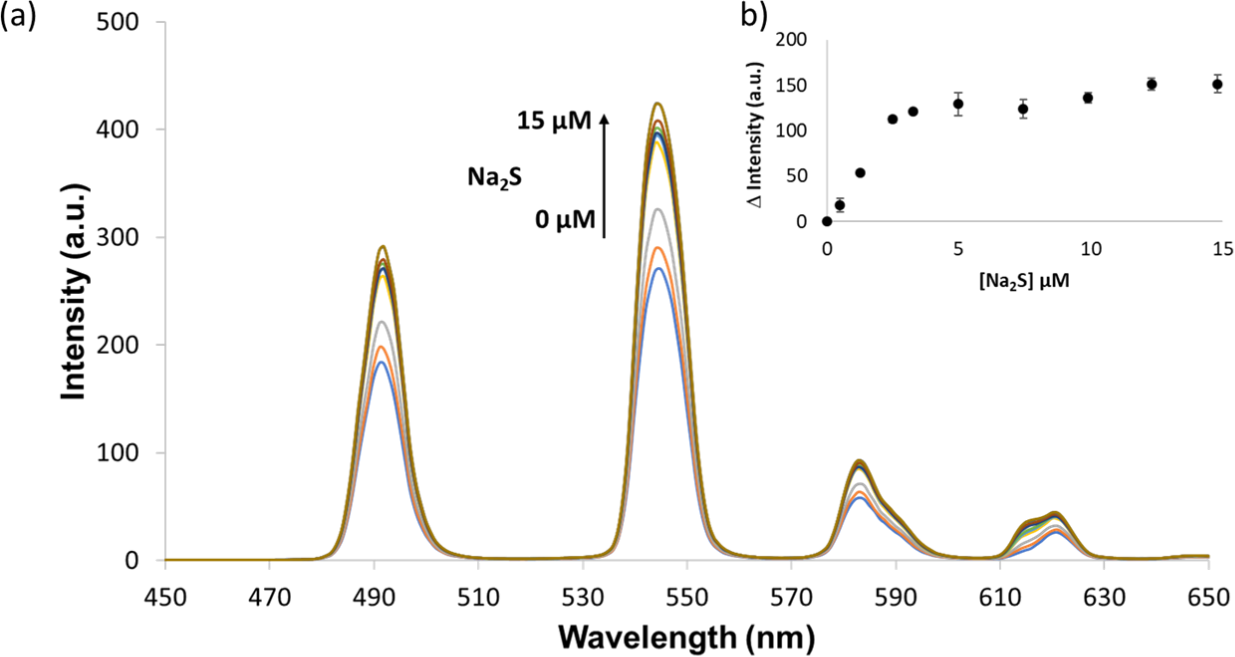
(a) Changes in the luminescence emission spectrum of 5 µM [Tb.**1**·3Cu]^3+^ upon the addition of Na_2_S (0–15 µM); spectra measured in 10 mM Tris-HCl buffer (pH 7.4) with λ_ex_ = 250 nm. (b) Change in luminescence intensity detected at 545 nm upon the addition of Na_2_S (*n* = 3).

**Figure 5:**
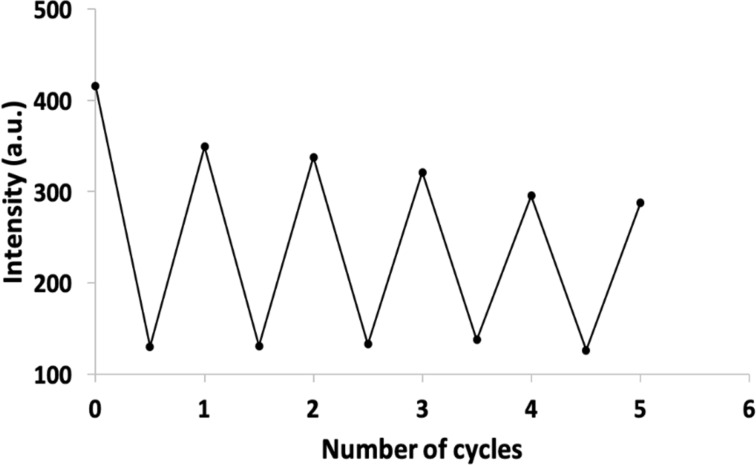
Luminescence intensity of 5 μM Tb.**1** in 10 mM Tris-HCl buffer, pH 7.4, containing 5% DMSO upon the alternate addition of Cu^2+^ ions (15 μM) ions and Na_2_S (15 μM); λ_ex_ = 250 nm, λ_em_ = 545 nm.

The addition of HS^−^ ions to [Tb.**1**·3Cu]^3+^ in Tris-HCl buffer resulted in a linear increase in luminescence over a concentration range of 0–15 μM (R^2^ = 0.974, Figure 4). The linear increase in luminescence, compared to the “lag-phase” observed in HEPES buffer, can be attributed to the Tris-HCl buffer forming a complex with Cu^2+^ ions, a phenomenon only weakly observed with HEPES buffer [19–20]. We hypothesize that using Tris-HCl buffer minimizes any formation of Cu(OH)_2_, facilitating the reaction of HS^−^_(aq)_ with [Tb.1·3Cu]^3+^, resulting in a linear rate of reaction. A 10-fold increase in luminescence regain was observed by the displacement of Cu^2+^ as CuS. Saturation in luminescence regain was observed after the addition of 3.0 equivalents of HS^−^_(aq)_ ions. The calculated theoretical LoD was 0.63 μM (20 ppb), which is comparable to that observed with the europium(III) analogue (1.1 μM, 36 ppb) [16]). The chemosensor exhibited significant reversibility over multiple cycles involving the addition and subsequent removal of Na_2_S followed by the precipitation and re-addition of Cu^2+^ ions (Figure 5).

**Figure 6:**
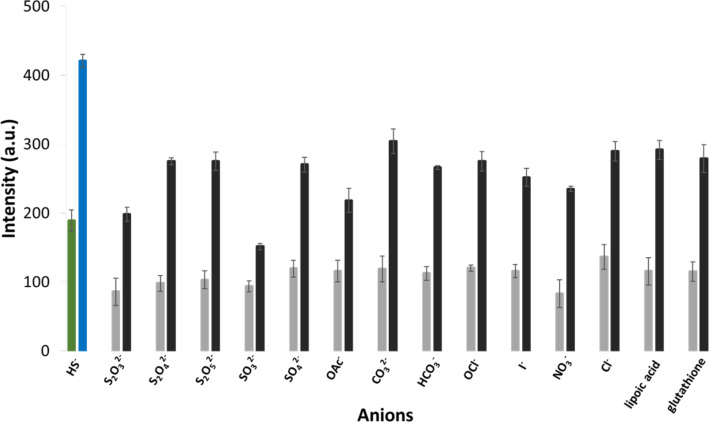
Changes in luminescent intensity of [Tb.**1**·3Cu]^3+^ (5 µM) detected at 545 nm in the presence of various anions/sulfur compounds; spectra measured in 10 mM Tris HCl buffer (pH 7.4) with λ_ex_ = 250 nm. Green bar: [Tb.**1**·3Cu]^3+^ alone (5 µM). Blue bar: [Tb.**1**·3Cu]^3+^ (5 µM) and Na_2_S (15 µM). Grey bars: [Tb.**1**·3Cu]^3+^ (5 µM) and anion/sulfur compound (50 µM). Black bars: [Tb.**1·**3Cu]^3+^ (5 µM), anion/sulfur compound (50 µM) and Na_2_S (15 µM), *n* = 3.

Comprehensive selectivity studies were conducted with various anions/sulfur compounds (SO_4_^2−^, SO_3_^2−^, S_2_O_5_^2−^, S_2_O_4_^2−^, S_2_O_3_^2−^, ClO^−^, OAc^−^, NO_3_^−^, I^−^, HCO_3_^−^, CO_3_^2−^, Cl^−^, lipoic acid, and glutathione, as depicted in Figure 6). It was interesting to note that neither of the sulfur-containing compounds caused a remarkable increase in luminescence, especially lipoic acid and glutathione which contain an –S–S (p*K*_a_ = 4.7) and –SH (p*K*_a_ = 9.65) group respectively similar to HS^−^_(aq)_ (p*K*_a_ = 6.9). This demonstrates that the sensors are highly selective to HS^−^_(aq)_ ions and are thus suitable for environmental or biological studies where interfering anions may be present.

### Gaseous H_2_S studies of [Tb.**1**·3Cu]^3+^

We aimed to further investigate the luminescence response of Tb^3+^ analogues upon exposure to hydrogen sulfide gas, building upon our previously reported findings. Our earlier work demonstrated an increase in europium luminescence with a LoD of 100 ppb and 665 ppb for two Eu analogues [Eu(triazole-DPA)_3_·3Cu]^3+^ [16] and [Eu(DO2A)DPA·Cu]^+^ [17], while the [Tb(DO2A)DPA·Cu]^+^ analogue [17] exhibited no significant change in luminescence upon exposure to gaseous H_2_S. To assess whether this observed behavior is consistent and potentially attributed to the energy levels of the Tb^3+^ ion, we conducted the gaseous studies with [Tb.**1**·3Cu]^3+^. Upon exposure to H_2_S gas using the established experimental setup, the [Tb.**1**·3Cu]^3+^ complex did not exhibit any discernible increase in luminescence.

**Figure 7:**
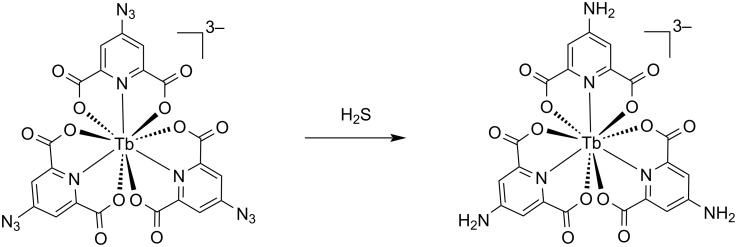
Chemical structure and mode of action of [Tb(DPA-N_3_)_3_]^3−^ for detection of H_2_S_(g)_ [11].

As far as we are aware, there are only three reports of lanthanide-based probes for the detection of gaseous hydrogen sulfide. Two are the europium(III) complexes from our group ([Eu(triazole-DPA)_3_·Cu]^3+^ and [Eu(DO2A)(triazole-DPA)·Cu]^+^), which are proposed to function by Cu^2+^ sequestration. The remaining report is of a terbium(III) complex [Tb(DPA-N_3_)_3_]^3−^ (Figure 7), which contains an aryl azide-functionalized ligand. In this system the azide functionality prohibits the energy transfer to the lanthanide ion, effectively quenching luminescence. In the presence of gaseous hydrogen sulfide, the aryl azide is reduced to an aniline functionality and luminescence is restored [11]. Drawing our previous findings and insights from the work of Hou, Wu, and co-workers [21], we postulate that gaseous H_2_S is interacting with the [Tb.**1**·3Cu]^3+^ complex as it did with ([Eu(triazole-DPA)_3_·Cu]^3+^, however for the terbium(III) complexes, the electronic state of the ligand, [triazole-DPA]^2−^ is altered, resulting in the energy gap of the ligand and the excited energy level of the Tb^3+^ ion being smaller. This would facilitate efficient back-energy transfer from the excited ^5^D_4_ level of the Tb^3+^ ion, a non-radiative process and consequently, explaining the absence of an observable change in luminescence for this complex.

## Conclusion

In conclusion, the Tb.**1** complex shows luminescent quenching in the presence of Cu^2+^ ions in both Tris-HCl and HEPES buffer. Luminescence is restored upon the addition of HS^−^_(aq)_ ions to [Tb.**1**·3Cu]^3+^, with a linear response observed until 1 equivalent of HS^−^_(aq)_ ions is added and Tris-HCl is used as the buffer (LoD 0.63 μM). Exposure of [Tb.**1**·3Cu]^3+^ to gaseous hydrogen sulfide did not result in an increase in the luminescent emission spectrum, unlike that observed with the europium(III) analogue. This result suggests potential variations in the luminescence response among terbium(III) analogues and highlights the complexity of the interaction between lanthanide complexes and gaseous H_2_S, and understanding the nuances of this interaction is the focus of our future research studies.

## Experimental

### Synthetic materials and methods

The Cu^2+^ ions were sourced from Cu(NO_3_)_2_·5H_2_O (Cat. #1027900250, Sigma-Aldrich). The synthesis of 4-(2-pyridyl)-1,2,3-triazole dipicolinic acid (**L**) was conducted as previously described [16]. Proton nuclear magnetic resonance (^1^H NMR) spectra was recorded on a Bruker DRX400 spectrometer operating at 400 MHz. High-resolution mass spectrometry (HRMS) was performed using a Bruker BioApex 47e FTMS with an analytical electrospray source employing NaI for accurate mass calibration (ESI). UV–vis absorption spectra were measured at room temperature utilizing a Varian Cary 1E UV–vis spectrophotometer with a quartz cell of 10 mm path length. Luminescence emission spectra of aqueous solutions were captured at 23 °C using a Varian Cary-Eclipse fluorescence spectrophotometer set to phosphorescence mode, employing a quartz cell with a 10 mm path length and a volume of 400 μL. The delay time was 0.1 ms, the gate time was 1 ms, and both the instrument's excitation and emission slit widths were set at 5 nm unless otherwise specified. Gaseous H_2_S was generated using an Advanced Calibration Designs (ACD) Cal 2000 calibration gas generator.

### Synthesis of Tb.**1**

To a solution of **L** (155 mg, 0.5 mmol) in water (6 mL) and 1 M NaOH (4 mL) a solution of Tb(OTf)_3_ (100 mg, 0.166 mmol) in water (2 mL) was added to obtain a white precipitate instantly. The solution was stirred at room temperature for a day. The precipitate was centrifuged, washed with water, and freeze-dried to obtain a white fluffy precipitate in 65% yield. HRESIMS (DMSO *m*/*z*): [Tb.**1**·2Na^+^]^−^ calcd. for C_42_H_21_TbN_15_O_12_Na_2_, 1132.0542; found, 1132.0583.

### Luminescence studies – analysis of aqueous solutions

This part was performed in a manner similar to that outlined in reference [17].

**Cu^2+^-dependent luminescence spectra**. A solution of Tb.**1** (5 μM) in 10 mM Tris–HCl buffer (pH 7.4, containing <5% DMSO), was incrementally spiked with a standard solution of 1 mM Cu(NO_3_)_2_·5H_2_O_(aq)_. The time-gated luminescence emission spectrum (λ_ex_ = 250 nm) of the solution was recorded after each addition.

**In situ preparation of the [Tb.1·3Cu]^3+ ^sensor**. Solutions of 5 μM [Tb.**1**·3Cu]^3+^ were prepared by combining a DMSO stock solution of Tb.**1** (100 μM) with 1 mM Cu(NO_3_)_2_·5H_2_O_(aq)_. Solutions were then diluted with the appropriate amount of buffer (final concentration 10 mM Tris–HCl or 10 mM HEPES buffer (pH 7.4), solutions contained <5% DMSO). The solutions were incubated at 23 °C for 5 min prior to use.

**Na_2_S-dependent luminescence spectra**. A solution of [Tb.**1**·3Cu]^3+^ (5 μM), in 10 mM buffer (final concentration 10 mM Tris–HCl or 10 mM HEPES buffer (pH 7.4), solutions contained <5% DMSO), was incrementally spiked with a standard solution of 1 mM Na_2_S_(aq)_. The time-gated luminescence emission spectrum (λ_ex_ = 250 nm) of the solution was recorded after each addition.

**Competition assay with anions and cations**: The time-gated luminescence emission change of a solution of [Tb.**1**·3Cu]^3+^ (5 μM), in 10 mM Tris–HCl buffer (pH 7.4, containing <5% DMSO), was examined in the absence and presence of 1.0 to 10.0 mol equiv of various anions. The anions were added as 1 mM aqueous solutions; NaCl, NaI, NaHCO_3_, Na_2_CO_3_, NaClO, NaNO_2_, NaOAc, Na_2_SO_3_, Na_2_SO_4_, Na_2_S_2_O_3_, Na_2_S_2_O_4_, Na_2_S_2_O_5_, lipoic acid, and glutathione. The change in luminescence emission spectra (λ_ex_ = 250 nm) of the solutions was also investigated after the subsequent addition of 1.0 molar equivalent of 1 mM Na_2_S_(aq)_.

**Quantum yields**. The quantum yield (φ) was determined using a quinine sulfate standard (φ = 0.55) for Tb·**1**, in water at pH 7.4 at 23 °C, according to the following equation:




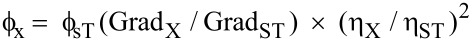




where the subscripts X and ST denote sample and standard, respectively, Grad is the gradient of plotted integrated luminescence intensity vs absorbance, and η is the refractive index of the solvent.

**Limit of detection (LoD)**. Solutions of [Tb.**1**·3Cu]^3+^ (5 μM) in 10 mM Tris–HCl buffer (pH 7.4, containing <5% DMSO) were incrementally spiked with a standard solution of 1 mM Na_2_S_(aq)_, with the time-resolved luminescence emission spectra recorded after each addition (λ_ex_ = 250 nm). From the measured data, the LoD was calculated according to the following equation; LoD = y_B_ + 3s_B_. y_B_ is the signal associated with the blank and s_B_ corresponds to the standard deviation of the blank [22–23]. LoD: [Tb.**1**·3Cu]^3+^ 0.63 μM, λ_em_ = 544 nm.

## Supporting Information

File 1 Copies of HRMS, ^1^H NMR and fluorescence emission spectra.

## Data Availability

All data that supports the findings of this study is available in the published article and/or the supporting information to this article.

## References

[1] Huang Y-Q, Jin H-F, Zhang H, Tang C-S, Du J-B, Zhu Y-C (2021). Interaction among Hydrogen Sulfide and Other Gasotransmitters in Mammalian Physiology and Pathophysiology. Advances in Hydrogen Sulfide Biology.

[2] Abe K, Kimura H (1996). J Neurosci.

[3] Liu D, Hessler W, Henary M (2023). Molecules.

[4] Cirino G, Szabo C, Papapetropoulos A (2023). Physiol Rev.

[5] Fosnacht K G, Pluth M D (2024). Chem Rev.

[6] Zhao Y, Biggs T D, Xian M (2014). Chem Commun.

[7] Letterman R D (1999). Water Quality and Treatment: A Handbook of Community Water Supplies.

[8] Thorson M K, Ung P, Leaver F M, Corbin T S, Tuck K L, Graham B, Barrios A M (2015). Anal Chim Acta.

[9] Tropiano M, Faulkner S (2014). Chem Commun.

[10] Yao Y, Kong C, Yin L, Jain A D, Ratia K, Thatcher G R J, Moore T W, Driver T G, Miller L W (2017). Chem – Eur J.

[11] Zhang R, Liu S, Wang J, Han G, Yang L, Liu B, Guan G, Zhang Z (2016). Analyst.

[12] Aulsebrook M L, Biswas S, Leaver F M, Grace M R, Graham B, Barrios A M, Tuck K L (2017). Chem Commun.

[13] Yao Y, Delgado-Rivera L, Samareh Afsari H, Yin L, Thatcher G R J, Moore T W, Miller L W (2018). Inorg Chem.

[14] Liang Z, Tsoi T-H, Chan C-F, Dai L, Wu Y, Du G, Zhu L, Lee C-S, Wong W-T, Law G-L (2016). Chem Sci.

[15] Aulsebrook M L, Graham B, Grace M R, Tuck K L (2018). Coord Chem Rev.

[16] Mini P, Springer M A, Grace M R, Dennison G H, Tuck K L (2020). Chem Commun.

[17] Mini P, Walker S E, Grace M R, Dennison G H, Tuck K L (2023). Dalton Trans.

[18] (2024). Online tools for supramolecular chemistry research and analysis.

[19] McPhail D B, Goodman B A (1984). Biochem J.

[20] Kotuniak R, Sudzik D Z, Ufnalska I M, Bal W (2024). Inorg Chem.

[21] Zeng X, Hu J, Zhang M, Wang F, Wu L, Hou X (2020). Anal Chem (Washington, DC, U S).

[22] Miller J C, Miller J N (1988). Statistics for analytical chemistry.

[23] Skoog D A, Holler F J, Crouch S R (2007). Principles of instrumental analysis.

