# Supplementary material for: Investigation of a bimetallic terbium(III)/copper(II) chemosensor for the detection of aqueous hydrogen sulfide
Source: Beilstein J Org Chem. 2024 Nov 5;20:2818–26. doi: 10.3762/bjoc.20.237 (PMC11552447; doi:10.3762/bjoc.20.237)
Supplement: File 1 — Copies of HRMS, 1H NMR and fluorescence emission spectra. [file Beilstein_J_Org_Chem-20-2818-s001.pdf]

BEILSTEIN JOURNAL OF ORGANIC CHEMISTRY

Supporting Information

for

Investigation of a bimetallic terbium(III)/copper(II) chemosensor for the detection of aqueous hydrogen sulfide

Parvathy Mini, Michael R. Grace, Genevieve H. Dennison and Kellie L. Tuck

Beilstein J. Org. Chem. **2024,** *20,* 2818–2826. doi:10.3762/bjoc.20.237

Copies of HRMS, ^{1}H NMR and fluorescence emission spectra

License and Terms: This is a supporting information file under the terms of the Creative Commons Attribution License (https://creativecommons.org/licenses/by/4.0). Please note that the reuse, redistribution and reproduction in particular requires that the author(s) and source are credited and that individual graphics may be subject to special legal provisions.
The license is subject to the *Beilstein Journal of Organic Chemistry* terms and conditions: (https://www.beilstein-journals.org/bjoc/terms)

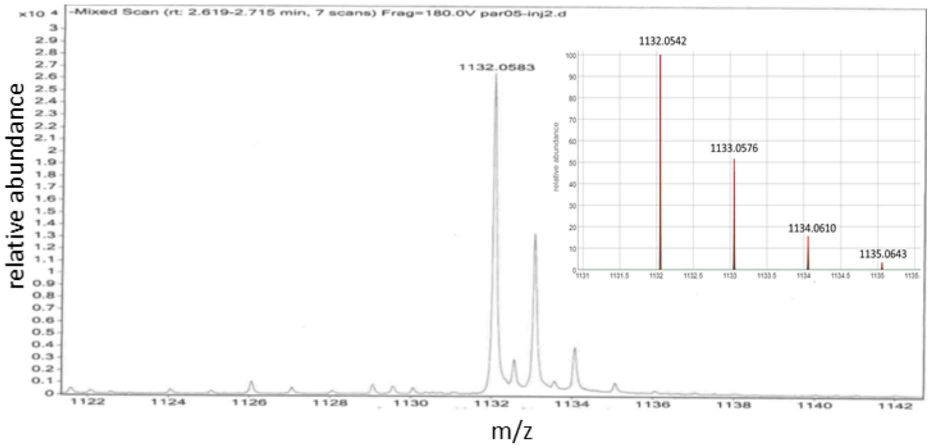

Figure S1: HRMS of [Tb.**1**·2Na^{+}]$^{-}$, insert: Simulated MS using EnviPat Web 2.4 software, available at https://www.envipat.eawag.ch/.

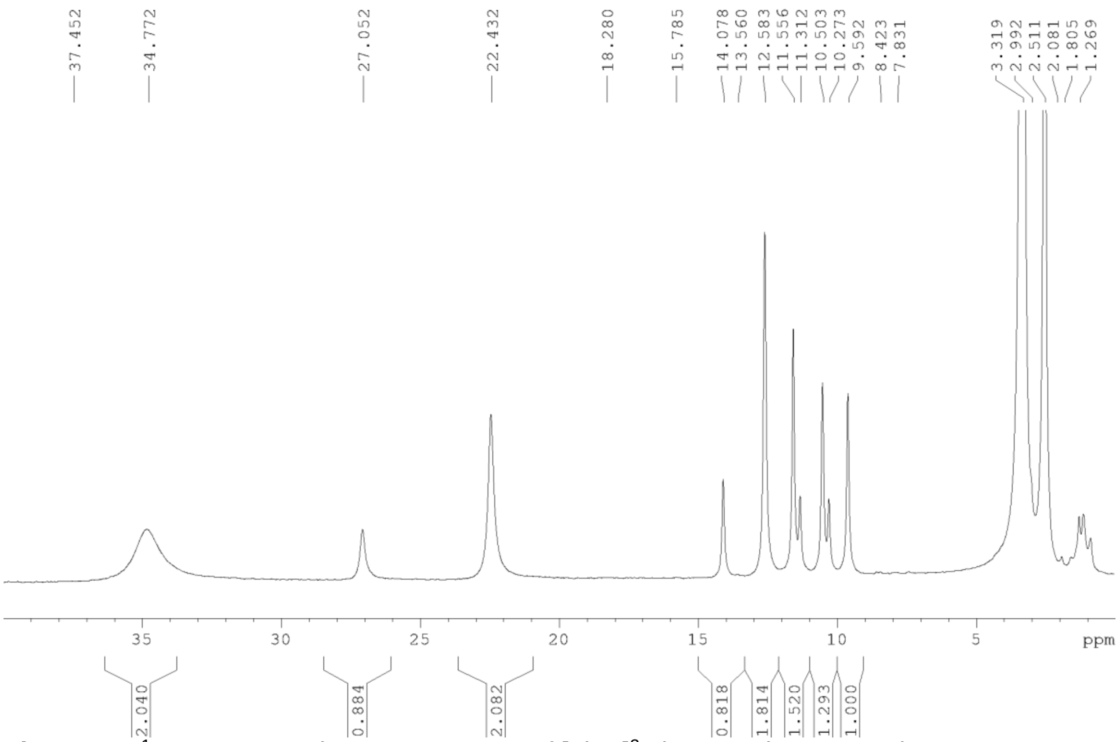

Figure S2. ^{1}H paramagnetic NMR spectrum of [Tb.**1**]$^{3-}$ (DMSO-d_6, 400 MHz).

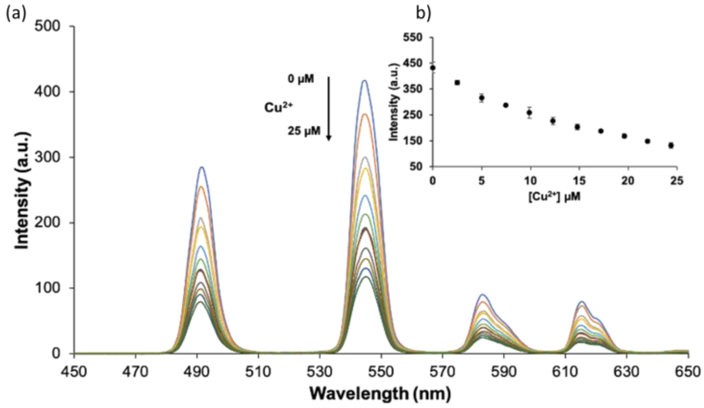

Figure S3: (a) Changes in the luminescence emission spectrum of [Tb.**1**]$^{3-}$ (5 μM) upon the addition of Cu^{2+} (0–25 μM); spectra measured in 10 mM Tris HCl buffer (pH 7.4) with λ_{ex} = 250 nm.

(b) Luminescence intensity detected at 545 nm upon the addition of Cu^{2+} (*n* = 3).

http://app.supramolecular.org/bindfit/view/d0e6f7ef-6faf-450b-b842-d8ae13300612

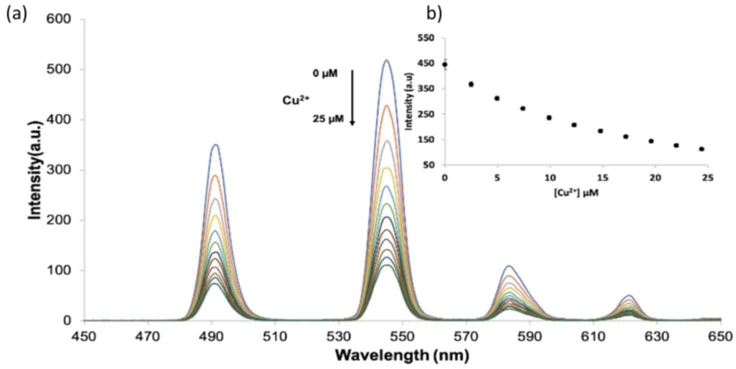

Figure S4: (a) Changes in the luminescence emission spectrum of [Tb.**1**]$^{3-}$ (5 μM) upon the addition of Cu^{2+} (0–25 μM); spectra measured in 10 mM HEPES buffer (pH 7.4) with λ_{ex} = 250 nm.

(b) Luminescence intensity detected at 545 nm upon the addition of Cu^{2+} (*n* = 3).

http://app.supramolecular.org/bindfit/view/9e883727-6b75-4a9f-835b-13a80df0e89d
